# Secondary Metabolites from *Sida rhombifolia* L. (Malvaceae) and the Vasorelaxant Activity of Cryptolepinone

**DOI:** 10.3390/molecules18032769

**Published:** 2013-03-01

**Authors:** Otemberg Souza Chaves, Roosevelt Albuquerque Gomes, Anna Cláudia de Andrade Tomaz, Marianne Guedes Fernandes, Leônidas das Graças Mendes Junior, Maria de Fátima Agra, Valdir Andrade Braga, Maria de Fátima Vanderlei de Souza

**Affiliations:** Postgraduate Program in Bioactive Natural and Synthetic Products, Health Sciences Center, Federal University of Paraíba, 58051-970, João Pessoa, PB, Brazil

**Keywords:** phytochemical study, *Sida rhombifolia* L., Malvaceae, cryptolepinone, vasorelaxant activity

## Abstract

The phytochemical study of *Sida rhombifolia* L. (Malvaceae) led to the isolation through chromatographic techniques of eleven secondary metabolites: sitosterol (**1a**) and stigmasterol (**1b**), sitosterol-3-*O*-β-d-glucopyranoside (**2a**) and stigmasterol-3-*O*-β-d-glucopyranoside (**2b**), phaeophytin A (**3**), 17^3^-ethoxypheophorbide A (**4**), 13^2^-hydroxy phaeophytin B (**5**), 17^3^-ethoxypheophorbide B (**6**), 5,7-dihydroxy-4'-methoxyflavone (**7**), cryptolepinone (**8**) and a salt of cryptolepine (**9**). Their structures were identified by ^1^H- and ^13^C-NMR using one- and two-dimensional techniques. In addition, the vasorelaxant activity of cryptolepinone in rat mesenteric artery rings is reported herein for the first time.

## 1. Introduction

The genus *Sida* has wide neotropical distribution, with several species well represented in the Americas. In Brazil this genus has many representatives in the Northeast and South and, to a lesser extent, in the North, Midwest and Southeast [1]. *Sida rhombifolia* (Malvaceae) is popularly known in Brazil as “matapasto”, “guanxuma” and “relógio”. It is used in Indian folklore medicine against hypertension, diabetes [2], as well as a traditional medicinal plant for the treatment of gout in Indonesia [3].

A variety of chemical constituents has been isolated from this species, including ecdysteroids [4] quinazoline alkaloids, besides β-phenylethylamines and carboxylated tryptamines [5]. The present study aimed to isolate and identify other classes of secondary metabolites from *S. rhombifolia* in order to associate them to pharmacological properties. We report the isolation and identification of two mixtures of steroids **1a**, **1b** and **2a** and **2b**, four porphyrins **3**, **4**, **5** and **6**, a flavone **7**, and two indoquinoline alkaloids **8** and **9**, besides showing for the first time the vasorelaxant activity of **8** in rat mesenteric artery with and without functional endothelium.

## 2. Results and Discussion

**Figure 1:**
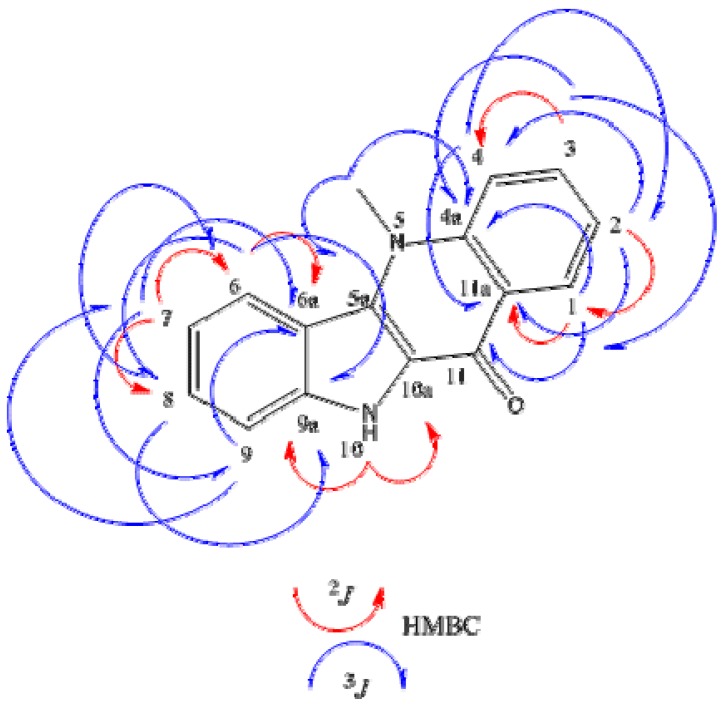
HMBC correlations of compound **8**.

**Figure 2:**
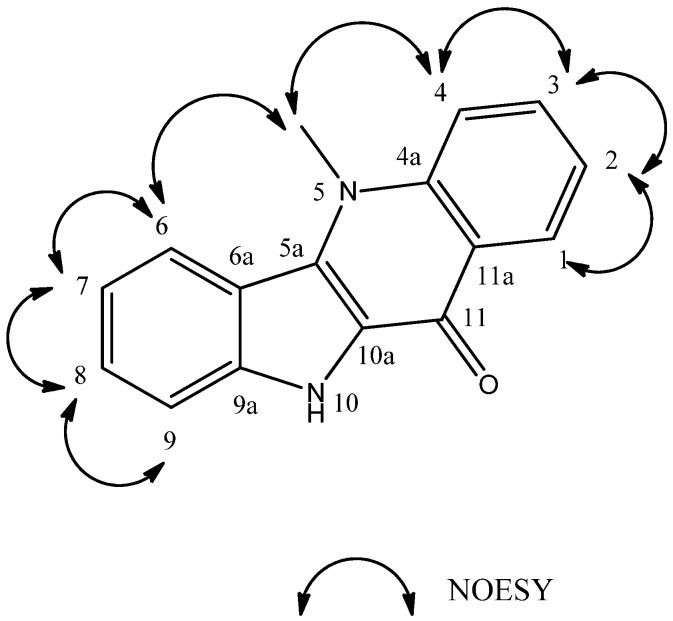
NOESY correlations of compound **8**.

Compound **8** appeared as yellow crystals with melting point >300 °C. The IR spectrum indicated the presence of NH (3431 cm^−1^) and carbonyl (1622 cm^−1^) groups. The ^1^H-NMR spectra of compound **8** (DMSO-*d*_6_) showed signals of indoquinoline-like alkaloids consisting of a double doublet [δ_H_ 8.44 (1H, dd, *J* = 8.1 and 1.4 Hz, H-1)], two doublet-doublet-triplets [δ_H_ 7.35 (1H, ddt, *J* = 8.1, 7.3 and 0.8 Hz, H-2)], 7.77 (1H, ddt, *J* = 8.7, 7.3 and 1.4 Hz, H-3)] and a broad doublet [δ_H_ 7.95 (1H, brd, *J* = 8.7 Hz, H-4)], characterizing the quinoline nucleus, besides two broad doublets [δ_H_ 8.38 (1H, brd, *J* = 8.4 Hz, H-6), 7.57 (1H, brd, *J* = 8.3 Hz, H-9)] and two other doublet-doublet-triplets [δ_H_ 7.47 (1H, ddt, *J* = 8.3, 7.6 and 1.0 Hz, H-8), 7.20 (1H, ddt, *J* = 8.4, 7.6 and 1.0 Hz, H-7)] characterizing the indole nucleus, thus leading to conclude that the indoquinoline nucleus was unsubstituted [6,7]. Furthermore, the spectra showed the presence of two singlets [δ_H_ 11.89 (1H, s), 4.36 (3H, s)] due to an indole NH and a methyl group bonded to nitrogen, respectively [6]. The ^13^C BB-NMR spectrum of **8** (DMSO-*d*_6_) showed 16 signals, consistent with an unsubstituted indoquinoline nucleus, being 15 aromatic carbons and one sp^3^-hybridized carbon. The presence of a methyl group bonded to nitrogen [δ_C_ 35.41] and a carbonyl [δ_C_ 166.45] could be highlighted. Based on the 2D NMR data of HMQC experiment the hydrogenated carbons found in ^13^C BB-NMR spectrum were established. The 2D NMR data of HMBC experiment showed the position of the nitrogen methyl in the quinoline nucleus due to the ^3^*J* correlation signal of the proton at δ_H_ 4.36 (s) to C-4a (δ_C_ 139.73) and C-5a (δ_C_ 129.89). The identity of hydrogen of the indole nucleus at δ_H_ 11.89 (s) was further confirmed by its ^2^*J* correlations to C-9a (δ_C_ 138.25) and C-10a (δ_C_ 123.03) and ^3^*J* correlations to C-6a (δ_C_ 115.70) and C-10a (δ_C_ 123.03) (Figure 1). The 2D NMR data of NOESY experiment (Figure 2) confirmed the position of the methyl at N-5 due to the correlations of the protons at δ_H-5_^1^ 4.36 (s) with the signals at δ_H-4_ 7.96 (d, *J* = 8.70 Hz) and δ_H-6_ 8.39 (d, *J* = 8.40 Hz) (Table 1). In addition, correlations between the protons from quinoline (H-1/H-2; H-2/H3; H-3/H-4) and indole nuclei (H-6/H-7, H-7/H-8; H-8/H-9) were established (Table 1) (Figure 1). Spectral data compilation and comparison of data in the literature [5] showed that **8** is cryptolepinone, a compound already isolated in the genus *Sida* [8], but isolated now for the first time from the species *Sida rhombifolia*.

**Table 1:** ^13^C-NMR (125 MHz), ^1^H-NMR (500 MHz), HMQC, HMBC and NOESY data of cryptolepinone (**8**) (DMSO-*d*_6_, δ ppm).

	HMQC		HMBC				NOESY
			^2^ *J* (H 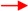 C)		^3^ *J* (H 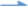 C)		(H↔H)
n°	δ_C_	δ_H_					
1	124.99	8.44 (dd, 1H, *J* = 8.1 and 1.4 Hz)	C-11a		C-11	C-4a	H-2
2	120.04	7.35 (ddt, 1H, *J =* 8.1; 7.3 and 0.8 Hz)	C-1		C-4	C-11a	H-1/H-3
3	130.71	7.77 (ddt, 1H, *J =* 8.7; 7.3 and 1.4 Hz)	C-4		C-1	C-4a	H-2/H-4
4	115.13	7.95 (brd, 1H, *J* = 8.7 Hz)			C-2	C-11a	H-3
4a	139.73	-					
5	-	-					
5a	129.89	-					
6	122.45	8.38 (brd, 1H, *J* = 8,4 Hz)	C-6a		C-8	C-9a	H-7
6a	115.70	-					
7	118.62	7.20 (ddt, 1H, *J =* 8.4; 7.6 and 1.0 Hz)	C-6	C-8	C-9	C-6a	H-6/H-8
8	126.54	7.47 (ddt, 1H *J =* 8.3; 7.6 and 1.0 Hz)			C-6	C-9a	H-7/H-9
9	112.36	7.57 (brd, 1H *J* = 8.3 Hz)			C-6a	C-7	H-8
9a	138.25	-					
10 (N-H)	-	11.89 (s, 1H)	C-10a	C-9a			
10a	123.03	-					
11	166.45	-					
11a	122.90	-					
N-CH_3_	35.41	4.36 (s, 3H)			C-4a	C-5a	H-4/H-6

**Figure 3:**
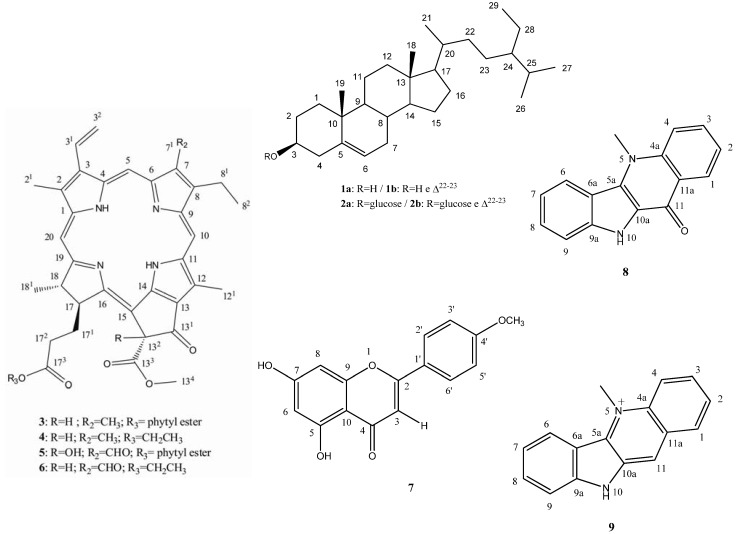
Chemical constituents isolated and identified from the crude ethanol extract of the aerial parts of *S. rhombifolia*. (Malvaceae).

Using similar methods as described above, compounds **1** to **7** and **9** were identified as sitosterol (**1a**) and stigmasterol (**1b**) [7], sitosterol-3-*O*-β-D-glucopyranoside (**2a**) and stigmasterol-3-*O*-β-D-glucopyranoside (**2b**) [9], phaeophytin A (**3**) [10], 17^3^-ethoxypheophorbide A (**4**) [1], 13^2^-hydroxy phaeophytin B (**5**) [11], 17^3^-ethoxypheophorbide **B** (**6**) [12], 5,7-dihydroxy-4'-methoxyflavone (acacetin, **7**) [13] and a salt of cryptolepine (**9**) [7] (Figure 3).

**Figure 4:**
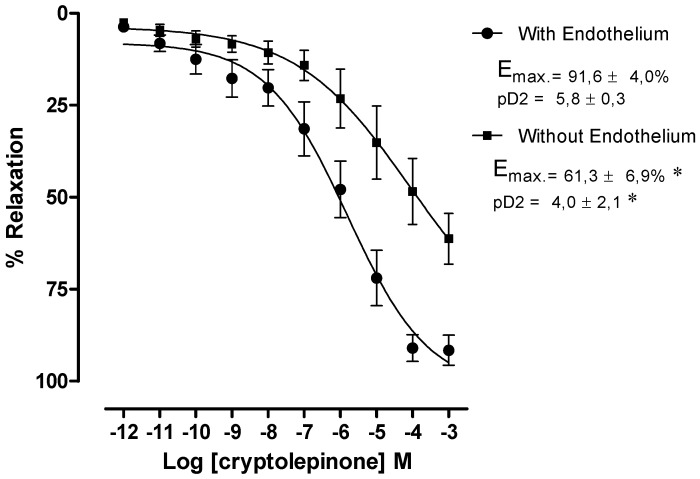
Concentration-response curve of **8** (10^−12^–10^−3^ M) in cranial mesenteric artery rings isolated from rats with functional endothelium (•) and without functional endothelium (■) pre-contracted with PHE (1 μM). Values are expressed as mean ± standard error of mean (SEM), * *p* < 0.05 compared to the ring with functional endothelium.

The vasorelaxant activity of **8** was evaluated by increasing cumulative addition of 10^−12^ to 10^−3^ M in rat cranial mesenteric artery rings pre-contracted with phenylephrine (PHE) at the concentration of 1 μM. It was observed that **8** caused a vasorelaxation in rings with functional endothelium (E_max_ = 91.6 ± 4.0%, n = 6) and after removal of endothelium, the vasorelaxant effect of **8** was significantly changed (61.3 ± 6.9%, n = 7) (Figure 4).

## 3. Experimental

### 3.1. General

Silica gel 60 (Merck) 7734 (0.063–0.2 mm particle, 70–230 mesh), flash silica (0.04–0.063 mm particles, 230–400 mesh), neutral alumina and Sephadex LH-20 were used for the fractionation and isolation of the secondary metabolites from *S. rhombifolia*. The melting point of the constituents was recorded on a MQAPF-302 apparatus (Microquímica Equipamentos Ltda., Palhoça-SC, Brazil). IR spectra were recorded on a FT-IR-1750 Perkin-Elmer spectrometer. ^1^H- and ^13^C-NMR spectra were recorded on a Varian Oxford 200 NMR spectrometer (200/50 MHz) and on a Varian 500 NMR spectrometer (500/125 MHz).

### 3.2. Collection, Extraction and Isolation

*Sida rhombifolia* was collected in the municipality of Santa Rita-PB in September 2008 and identified by Dr. Maria de Fátima Agra. A voucher specimen is kept at the herbarium Professor Lauro Pires Xavier (CCEN/UFPB) under the code Agra 7045. The dried and powdered aerial parts of the plant (5.5 kg) were extracted with 95% EtOH (10 L, room temperature) for 72 h. The EtOH extract was concentrated under reduced pressure at 40 °C, providing 570 g of crude ethanol extract (CEE). Part of the CEE (200.0 g) was subjected to filtration under reduced pressure using silica gel 60 as stationary phase and eluted with hexane (Hex.), ethyl acetate (EtOAc) and methanol (MeOH) alone or in binary mixtures following an increasing gradient polarity. The fractions obtained were concentrated under reduced pressure. Fraction Hex.–AcOEt (9:1) (6.0 g) was subjected to silica gel 60 column chromatography (CC) eluted with Hex., dichloromethane (CH_2_Cl_2_) and MeOH alone or in binary mixtures following an increasing gradient polarity, yielding 35 mg of compound **1**. Fraction Hex.–AcOEt (7:3) (6.11 g) was subjected to the same methodology, but using as eluents Hex., EtOAc and MeOH. A fraction of this column was subjected to preparative TLC in Hex.–EtOAc (9:1) to give 35 mg of **3**. Then, the initial TLC band was rechromatographed in Hex.–EtOAc (8:2) to give 5.0 mg of compound **5**. Compound **2** (33.0 mg) was obtained from the fraction EtOAc (1.23 g) using the previous technique for column chromatography. Fraction EtOAc–MeOH (9:1) yielded a precipitate and a supernatant (1.24 g). The latter was subjected to CC on Sephadex LH-20 using methanol as eluent. This procedure yielded 10.0 mg of compound **7**. Another part of the CEE (200.0 g) was dissolved in EtOH–H_2_O (9:1) and subjected to liquid-liquid chromatography using Hex., CH_2_Cl_2_, EtOAc and *n*-butanol, obtaining the respective phases, besides the hydroalcoholic phase. The CH_2_Cl_2_ phase (26.0 g) was subjected to filtration under reduced pressure with the solvents Hex., CH_2_Cl_2_ and MeOH. The fractions Hex.–CH_2_Cl_2_ (3:7) (17.4 mg) and CH_2_Cl_2_ (200.0 mg) were combined and subjected to CC using flash silica as adsorbent and the eluents Hex., EtOAc and MeOH alone or in binary mixtures following an increasing gradient polarity. Compounds **4** (25.0 mg) and **6** (15.0 mg) were thus obtained. The CEE of *Sida rhombifolia* reacted positively with Dragendorff, Bouchadart and Mayer reagents, as expected according to literature. The classic alkaloid extraction was performed with 100 g of the CEE. The total alkaloid content (800 mg) was subjected to CC on neutral alumina and the elution system was the same of the previous methodology. Compounds **8** (28.0 mg) and **9** (18.0 mg) were isolated.

*Phaeophytin A* (**3**) (without phytyl ester): Green powder. IR (KBr) v_max_: 3435, 2926, 2854, 1735, 1701, 1618, 1377 cm^−1^. ^1^H-NMR (500.00 MHz) (CDCl_3_) δ: 3.37 (s, 3H, H-2^1^), 7.92 (1H, dd, *J* = 17.3 and 11.2 Hz, H-3^1^), 6.25 and 6.15 (2H, d and d, *J* = 17.3 and 11.2 Hz, H-3^2^), 9.28 (s, 1H, H-5), 3.15 (s, 1H, H-7^1^), 3.60 (m, 2H, H-8^1^), 1.66 (m, 3H, H-8^2^), 9.44 (s, 1H, H-5), 3.68 (s, 3H, H-12^1^), 6.29 (s, 1H, H-13^2^), 3.91 (s, 3H, H-13^4^), 4.04 (m, 1H, H-17), 1.14 (m, 2H, 17^1^), 4.30 (m, 1H, H-18), 1.85 (3H, d, *J* = 7.3 Hz, H-18^1^), 8.57 (s, 1H, H-20). ^13^C-NMR (125.00 MHz) (CDCl_3_) δ: 142.26 (C-1), 131.08 (C-2), 12.26 (C-2^1^), 136.69 (C-3), 129.21 (C-3^1^), 122.95 (C-3^2^), 136.69 (C-4), 97.65 (C-5), 155.58 (C-6), 136.34 (C-7), 11.33 (C-7^1^), 145.30 (C-8), 19.57 (C-8^1^), 17.54 (C-8^2^), 149.93 (C-9), 104.72 (C-10), 138.11 (C-11), 128.46 (C-12), 12.29 (C-12^1^), 128.12 (C-13), 189.83 (C-13^1^), 64.93 (C-13^2^), 173.14 (C-13^3^), 53.20 (C-13^4^), 149.93 (C-14), 105.48 (C-15), 161.17 (C-16), 51.55 (C-17), 29.91 (C-17^1^), 31.40 (C-17^2^), 173.23 (C-17^3^), 50.33 (C-18), 23.38 (C-18^1^), 172.46 (C-19), 93.41 (C-20).

*17^3^-Ethoxypheophorbide A* (**4**): Bluish green powder with a metallic luster. ^1^H-NMR (500.00 MHz) (CDCl_3_) δ: 3.31 (s, 3H, H-2^1^), 7.77 (1H, dd, *J* = 17.5 and 12.5 Hz, H-3^1^), 6.14 and 6.05 (2H, d and d, *J* = 17.5 and 12.5Hz, H-3^2^), 9.07 (s, 1H, H-5), 2.98 (s, 3H, H-7^1^), 3.41 (2H, q, *J* = 15.0 Hz, H-8^1^), 1.58 (3H, t, *J* = 10.0 Hz, H-8^2^), 9.28 (s, 1H, H-10), 3.63 (s, 3H, H-12^1^), 6.30 (s, 1H, H-13^2^), 3.93 (s, 3H, H-13^4^), 4.24 (1H, brd, *J* = 10.0 Hz, H-17), 1.27 (brs, 2H, H-17^1^), 2.22–2.39 (2H, m, H-17^2^), 4.07 (2H, q, *J* = 12.5 Hz, H-17^4^), 1.15 (3H, t, *J* = 5.0 Hz, H-17^5^), 4.48 (m, 1H, H-18), 1.85 (3H, d, *J* = 5.0 Hz, H-18^1^), 8,53 (s, 1H, H-20). ^13^C-NMR (125.00 MHz) (CDCl_3_) δ: 141.93 (C-1), 131.66 (C-2), 11.93 (C-2^1^), 135.90 (C-3), 128.83 (C-3^1^), 122.47 (C-3^2^), 135.94 (C-4), 97.24 (C-5), 155.37 (C-6), 135.90 (C-7), 10.89 (C-7^1^), 144.88 (C-8), 19.14 (C-8^1^), 17.22 (C-8^2^), 150.74 (C-9), 104.12 (C-10), 137.78 (C-11), 128.80 (C-12), 11.98 (C-12^1^), 128.80 (C-13), 189.58 (C-13^1^), 64.72 (C-13^2^), 172.89 (C-13^3^), 52.80 (C-13^4^), 149.60 (C-14), 105.14 (C-15), 161.17 (C-16), 51.16 (C-17), 29.82 (C-17^1^), 31.26 (C-17^2^), 172.11 (C-17^3^), 60.48 (C-17^4^), 14.06 (C-17^5^), 50.10 (C-18), 23.04 (C-18^1^), 169.61 (C-19), 92.98 (C-20).

*13^2^-Hydroxy phaeophytin B* (**5**) (without phytyl ester): Yellow green powder. IR (KBr) v_max_: 3435, 1161 cm^−1^. ^1^H-NMR (200.00 MHz) (CDCl_3_) δ: 3.59 (s, 3H, H-2^1^), 8.02 (1H, dd, *J =* 17.5 and 11.6 Hz, H-3^1^), 6.37 and 6.22 (2H, d and d, *J* = 17.5 and 11.6 Hz, H-3^2^), 9.76 (s, 1H, H-5), 11.17 (s, 1H, H-7^1^), 4.10 (2H, brd, *J* = 7.8 Hz, H-8^1^), 1.83 (3H, t, *J* = 7.5 Hz, H-8^2^), 10.47 (s, 1H, H-10), 3.71 (s, 1H, H-12^1^), 3.85 (s, 3H, H-13^4^), 5.31 (1H, brd, *J* = 3.4 Hz, H-17), 4.41 (m, 1H, H-18), 1.57 (3H, d, *J* = 7.6 Hz, H-18^1^), 8.58 (s, 1H, H-20).

*17^3^-Ethoxypheophorbide **B*** (**6**): Yellow green powder with a metallic luster. IR (KBr) v_max_: 2729 cm^−1^. ^1^H-NMR (500.00 MHz) (CDCl_3_) δ: 3.36 (s, 3H, H-2^1^), 7.97 (1H, dd, *J* = 15.0 and 10.0 Hz, H-3^1^), 6.20 and 6.23 (2H, d and d, *J* = 15.0 and 10.0 Hz, H-3^2^), 10.33 (s, 1H, H-5), 11.12 (s, 1H, H-7^1^), 4.00 (m, 2H, H-8^1^), 1.62 (m, 3H, H-8^2^), 9.61 (s, 1H, H-10), 3.66 (s, 3H, H-12^1^), 6.21 (s, 1H, H-13^2^), 3.88 (s, 3H, H-13^4^), 4.44 (1H, brd, *J* = 10.0 Hz, H-17), 2.46–2.61 (m, 2H, H-17^1^), 2.20–2.33 (m, 2H, H-17^2^), 4.00 (m, 2H, H-17^4^), 1.10 (3H, t, *J* = 7.5 Hz, H-17^5^), 4.18 (1H, brd, *J* = 10.0 Hz, H-18), 1.80 (3H, d, *J* = 5.0 Hz, H-18^1^), 8.52 (s, 1H, H-20). ^13^C-NMR (125.00 MHz) (CDCl_3_) δ: 143.57 (C-1), 132.44 (C-2), 12.05 (C-2^1^), 135.54 (C-3), 128.65 (C-3^1^), 123.57 (C-3^2^), 137.19 (C-4), 101.60 (C-5), 159.38 (C-6), 137.19 (C-7), 187.72 (C-7^1^), 147.19 (C-8), 19.13 (C-8^1^), 19.36 (C-8^2^), 150.73 (C-9), 105.01 (C-10), 138.04 (C-11), 129.75 (C-12), 12.23 (C-12^1^), 132.20 (C-13), 189.49 (C-13^1^), 64.58 (C-13^2^), 172.76 (C-13^3^), 52.95 (C-13^4^), 151.25 (C-14), 104.96 (C-15), 164.03 (C-16), 51.35 (C-17), 31.25 (C-17^1^), 29.70 (C-17^2^), 174.00 (C-17^3^), 60.55 (C-17^4^), 14.07 (C-17^5^), 50.12 (C-18), 23.04 (C-18^1^), 169.25 (C-19), 93.55 (C-20).

*5,7-Dihydroxy-4'-methoxyflavone* (**7**): Yellow crystals. ^1^H-NMR (500.00 MHz) (CD_3_OD) δ: 6.63 (s, 1H, H-3), 6.21 (1H, d, *J* = 1.75 Hz, H-6), 6.46 (1H, d, *J* = 1.75 Hz, H-8), 7.94 (2H, d, *J* = 9.0 Hz, H-2’/H-6’), 7.08 (2H, d, *J* = 9.0 Hz, H-3'/H-6'), 3.88 (s, 3H, OCH_3_ H-4'). ^13^C-NMR (125.00 MHz) (DMSO-d_6_) δ: 163.73 (C-2), 102.99 (C-3), 181.21 (C-4), 160.87 (C-5), 98.37 (C-6), 162.75 (C-7), 93.49 (C-8), 156.79 (C-9), 103.19 (C-10), 122.30 (C-1’), 127.74 (C-2'/C-6'), 114.04 (C-3'/C-5'), 161.76 (C-4'), 55.02 (OCH_3_ C-4'). 

*Cryptolepinone* (**8**): Yellow crystals. IR (KBr) v_max_: 3,431, 1,622 cm^−1^. ^1^H-NMR and ^13^C-NMR: See Table 1.

*Cryptolepine* (**9**): Yellow crystals. ^1^H-NMR (500.00 MHz) (CD_3_OD) δ: 8.45 (1H, dd, *J* = 8.3 and 1.5 Hz, H-1), 7.91 (2H, m, H-2/H-8), 8.16 (1H, ddd, *J* = 9.1, 6.9 and 1.5 Hz, H-3), 8.63 (1H, brd, *J* = 9.1 Hz, H-4), 8.71 (1H, brd *J* = 8.5 Hz, H-6), 7.53 (1H, ddd, *J* = 8.5 , 7.1 and 1.0 Hz, H-7), 7.77 (1H, dd, *J* = 8.4 and 1.0 Hz, H-9), 9.11 (s, 1H, H-11), 5.07 (s, 3H, CH_3_ N-5). ^13^C-NMR (125.00 MHz) (CD_3_OD) δ: 131.10 (C-1), 128.50 (C-2), 134.00 (C-3), 118.30 (C-4), 139.80 (C-5a), 126.80 (C-6), 115.30 (C-6a), 123.00 (C-7), 135.40 (C-8), 114.30 (C-9), 147.70 (C-9a), 135.00 (C-10a), 126.10 (C-11), 128.00 (C-11a), 40.7 (CH_3_ N-5).

### 3.3. Bioactivity Assay

Experimental procedures were performed as described by França-Silva *et al.* [14] and were approved by the Federal University of Paraíba Ethical Committee for Animal Use under the protocol CEPA#305/09. After euthanasia by decaptation using a guilliotine, the cranial mesenteric artery was isolated, placed in Tyrode’s solution and dissected in order to make it free of adhering tissue. In endothelium-denuded experiments, endothelium was removed by rubbing the intimal surface of the vessels. Rings with 1–2 mm were obtained and placed in physiological Tyrode’s solution, maintained to 37 °C, gassed with carbogenic mixture (95% O_2_ and 5% CO_2_) and kept at pH 7.4. All preparations were stabilized under a resting tension of 0.75 g for 1 h. The solution was replaced every 15 min in order to prevent the accumulation of metabolites. The force of contraction was isometrically recorded by a force transducer (Miobath-4, WPI, Sarasota, FL, USA) coupled to an amplifier-recorder (Miobath-4) and to a computer equipped with an analog–to–digital converter board as described earlier. The presence of functional endothelium was assessed by the ability of acetylcholine (10 µM) to induce more than 80% relaxation of vessels pre-contracted with phenylephrine (10 µM). Less than 10% of relaxation to acetylcholine was taken as evidence that the vessel segments were functionally denuded of endothelium. The preparations were exposed to cumulatively concentrations of cryptolepinone (10^−12^–10^−3^ M) added to preparations until a maximum response to the drug accumulation was observed as indicated by a plateau response (approximately 3–5 min).

## 4. Conclusions

The phytochemical study of the crude ethanol extract from the aerial parts of *Sida rhombifolia* (Malvaceae) led to the identification of eleven compounds: two mixtures of steroids (**1a** and **1b**; **2a** and **2b**), four porphyrins (**3**, **4**, **5** and **6**), a flavone (**7**), and two indoquinoline alkaloids (**8** and **9**).

The compounds 13^2^-hydroxyphaeophytin B (**5**) and 17^3^-ethoxypheophorbide B (**6**) are being described hering for the fiorst time in the family Malvaceae, and the flavonoid 5,7-dihydroxy-4'-methoxyflavone (acacetin, **7**) is reported herein for the first time in the genus *Sida* and the other compounds were isolated for the first time in the species *S. rhombifolia*. In addition, the vasorelaxant activity of cryptolepinone (**8**) in rat cranial mesenteric artery with and without functional vascular endothelium is herein reported for the first time.

## References

[1] Silva D.A., Silva T.M.S., Lins A.C.S., Costa D.A., Cavalcante J.M.S., Matias W.N.,  Souza M.F.V., Braz Filho R. (2006). Constituintes Químicos e Atividade Antioxidante de *Sida galheirensis* Ulbr. (Malvaceae). Quim. Nova.

[2] Thounaojam M.C., Jadeja R.N., Ansarullah, Patel V.B., Devkar R.V., Ramachandran A.V. (2009). Potential of *Sida rhomboidea.Roxb* Leaf Extract in Controlling Hypertriglyceridemia in Experimental Models. Pharmacognosy Res..

[3] Iswantini D., Darusman L.K., Hidayat R. (2009). Indonesian *Sidaguri* (*Sida rhombifolia* L.) as Antigout and Inhibition Kinetics of Flavonoids Crude Extract on the Activity of Xanthine Oxidase. J. Biol. Sci..

[4] Jadhava A.N., Pawara R.S., Avulaa B., Khan I.A. (2007). Ecdysteroid Glycosides from *Sida rhombifolia* L.. Chem. Biodivers..

[5] Prakash A., Varma R.K., Ghosal S. (1981). Chemical Constituents of the Malvaceae. Part III. Alkaloidal Constituents of *Sida acuta*, *Sida humilis*, *Sida rhombifolia* and *Sida spinosa*. Planta Med..

[6] Martin G.E., Guido J.E., Robins R.H., Sharaf M.H.M., Schiff P.L., Tackie A.N. (1998). Submicro Inverse-Detection Gradient NMR: A Powerful New Way of Conducting Structure Elucidation Studies with <0.05 micromol Samples. J. Nat. Prod..

[7] Tousek J., Vanmiert S., Pieters L., Baelen G.V., Hostyn S., Maes B.U.W., Lemiere G., Dommisse R., Marek R. (2008). Structural and Solvent Effects on the ^13^C and ^15^N NMR Chemical Shifts of Indoloquinoline Alkaloids: Experimental and DFT Study. Magn. Reson. Chem..

[8] Karou S.D., Nadembega W.M.C., Ilboudo D.P., Ouermi D., Gbeassor M., de Souza C., Simpore J. (2007). *Sida acuta* Burm. f.: A Medicinal Plant with Numerous Potencies. Afr. J. Biotechnol..

[9] Kojima H., Sato N., Hatano A., Ogura H. (1990). Sterol Glucosides from *Prunella vulgaris*. Phytochemistry.

[10] Tomaz A.C.A., Nogueira R.B.S.S., Pinto D.S., Agra M.F., Souza M.F.V., Da-Cunha E.V.L. (2008). Chemical Constiuents from *Richardia grandiflora* (Cham. & Schltdl.) Steud. (Rubiaceae). Rev. Bras. Farmacogn..

[11] Sakdarat S., Shuyprom A., Ayudhya T.D., Waterman P.G., Karagianis G. (2006). Chemical Composition Investigation of the *Clinacanthus nutans* Lindau Leaves. Thai J. Phytopharm..

[12] Chee C-F, Lee H.B., Ong H.C., Siong-Hockho A. (2005). Photocytotoxic Pheophorbide-Related Compounds from *Aglaonema simplex*. Chem. Biodivers..

[13] Gomes R.A., Ramirez R.R.A., Maciel J.K.S., Falcão-Siva V.F., Siqueira-Junior J.P., Agra M.F., Souza M.F.V. (2011). Phenolic compounds from *Sidastrum micranthum* (A. St.-Hil.) Fryxell and Evaluation of Acacetin and 7,4'-Di-*O*-Methylisoscutellarein as Modulator of Bacterial Drug Resistance. Quim. Nova.

[14] França-Silva M.S., Luciano M.N., Ribeiro T.P., Silva J.S., Santos A.F., França K.C., Nakao L.S., Athayde-Filho P.F., Braga V.A., Medeiros I.A. (2012). The 2-Nitrate-1,3-Dibuthoxypropan, a New Nitric Oxide Donor, Induces Vasorelaxation in Mesenteric Arteries of the Rat. Eur. J. Pharmacol..

